# Strategic Interviewing to Detect Deception: Cues to Deception across Repeated Interviews

**DOI:** 10.3389/fpsyg.2016.01702

**Published:** 2016-11-01

**Authors:** Jaume Masip, Iris Blandón-Gitlin, Carmen Martínez, Carmen Herrero, Izaskun Ibabe

**Affiliations:** ^1^Department of Social Psychology and Anthropology, University of SalamancaSalamanca, Spain; ^2^Department of Psychology, California State University Fullerton, FullertonCA, USA; ^3^Department of Social Psychology and Methodology of the Behavioral Sciences, University of the Basque CountrySan Sebastián, Spain

**Keywords:** deception detection, lie detection, inconsistency, strategic interviewing, cognitive load, alibi, deception cues

## Abstract

Previous deception research on repeated interviews found that liars are not less consistent than truth tellers, presumably because liars use a “repeat strategy” to be consistent across interviews. The goal of this study was to design an interview procedure to overcome this strategy. Innocent participants (truth tellers) and guilty participants (liars) had to convince an interviewer that they had performed several innocent activities rather than committing a mock crime. The interview focused on the innocent activities (alibi), contained specific central and peripheral questions, and was repeated after 1 week without forewarning. Cognitive load was increased by asking participants to reply quickly. The liars’ answers in replying to both central and peripheral questions were significantly less accurate, less consistent, and more evasive than the truth tellers’ answers. Logistic regression analyses yielded classification rates ranging from around 70% (with consistency as the predictor variable), 85% (with evasive answers as the predictor variable), to over 90% (with an improved measure of consistency that incorporated evasive answers as the predictor variable, as well as with response accuracy as the predictor variable). These classification rates were higher than the interviewers’ accuracy rate (54%).

## Introduction

In 2000, Jonathan Kaled, Matthew Daniels, and Frank Kuecken, teenagers from New Baltimore, Michigan, were arrested and charged with the murder of Justin Mello during the robbery of the pizza place where Mello worked. Kaled and Kuecken confessed to the crime, but they later recanted, arguing that they had been coerced into confessing by the police. However, “[i]n light of the confessions, claims of individuals that the three men had all been present at the same party miles away from New Baltimore at the time of the murder were not taken seriously” (Drizin and Leo, 2004, p. 978). Charges against Daniels were later dropped, but Kaled and Kuecken spent over 6 months in prison before being released after the real perpetrator confessed (Drizin and Leo, 2004). In this paper, we present the first steps toward developing an interview procedure that could be useful to assess the veracity of suspects in cases such as Kaled, Daniels, and Kuecken’s.

### The Challenge to Detect Deception in Criminal Investigations: Inconsistencies and Deception

There are contexts where detecting deception is crucial. For instance, law enforcement officers need to be able to ascertain whether suspects denying their criminal involvement are lying or telling the truth. King and Dunn (2010) stated that:

*[L]ying to the police and other law enforcement officials can have profound judicial consequences. The detection of deception may greatly concern those in law enforcement, including police officers collecting evidence, customs officials attempting to prevent terrorists from crossing a border, detectives interrogating a suspect, and intelligence operatives pressing for information concerning potential attacks. Deception in these settings may take a number of forms. For example, a criminal may lie in order to avoid further scrutiny or arrest. Witnesses may lie to either avoid providing pertinent information or to fabricate information. Similarly, victims may embellish their accounts or withhold crucial information. Any of these deceptions may thwart a law enforcement investigation* (p. 306).

However, detecting deception is extremely difficult. Meta-analyses reveal that the humans’ ability to separate truths from lies on the basis of the sender’s behavior hardly exceeds chance probability (Aamodt and Custer, 2006; Bond and DePaulo, 2006), and that practitioners whose jobs require skill at detecting deception are not more accurate than lay people (e.g., Aamodt and Custer, 2006; Bond and DePaulo, 2006; Vrij, 2008). The reason for this low performance is that behavioral differences between truth tellers and liars are small and may change under the influence of a number of moderator variables (DePaulo et al., 2003; Sporer and Schwandt, 2006, 2007). In a series of meta-analyses, Hartwig and Bond (2011) found that humans trying to judge veracity pay attention to the right cues, but even those cues are not very strongly associated with veracity (Hartwig and Bond, 2011). As a result, accuracy rates are low. The corollary is clear: to improve observers’ detection accuracy, behavioral differences between truth tellers and liars should be increased (Hartwig and Bond, 2011).

Over the last decade, researchers have been working toward this goal. A number of interviewing approaches have been developed to increase behavioral differences between liars and truth tellers (see reviews by Vrij et al., 2010a; Vrij and Granhag, 2012; Vrij, 2014). Some of these approaches attempt to elicit inconsistencies in liars but not in truth tellers (see Vredeveldt et al., 2014).

Different interviewing approaches can produce different sorts of inconsistencies. It may be useful to differentiate between statement-evidence, between-person, and within-person inconsistencies. The Strategic Use of Evidence Technique (see, e.g., Granhag et al., 2007; Hartwig et al., 2014) aims at eliciting inconsistencies between the guilty suspects’ statements and the evidence available to the police (*Statement-evidence inconsistencies*). The unanticipated questions approach was designed by Vrij et al. (2009) to detect inconsistencies between pairs of liars interviewed separately and asked questions they could hardly have anticipated—so they were not able to agree on a common answer before the interview. These are *between-person inconsistencies*. Finally, Leins et al. (2011, 2012) designed a procedure to generate inconsistencies between the liars’ sketch drawings of the layout of a restaurant (Leins et al., 2011) or a room (Leins et al., 2012) and their own verbal description of these places. These are *within-person inconsistencies*. Here we describe the first steps toward creating a new interview approach that attempts to generate another variant of within-person inconsistencies: discrepancies between separate statements of the same individual.

Previous research examining within-person inconsistencies across repeated interviews revealed that liars were *not* less consistent than truth tellers. Instead, liars were as or more consistent than truth tellers (see Granhag and Strömwall, 2000, 2001, 2002; Granhag et al., 2003; Strömwall and Granhag, 2005). The authors explained this finding in terms of the *repeat versus reconstruct hypothesis* (Granhag and Strömwall, 1999). Drawing on the notion that liars might try to manipulate their behavior in order to look honest (e.g., Buller and Burgoon, 1996; Zuckerman et al., 1981), Granhag and Strömwall (1999) reasoned that liars actively (and successfully) tried not to contradict themselves across repeated interviews. Conversely, truth tellers simply tried to recall the original experience in all interviews. However, because human memory is reconstructive and error prone (e.g., Tulving, 2000; Loftus, 2003), the truth tellers’ recollections showed some discrepancies (Granhag and Strömwall, 1999; Granhag et al., 2003; Strömwall and Granhag, 2005). As a result, the net amount of inconsistencies was very similar regardless of veracity.

These findings do not imply that liars and truth tellers cannot be differentiated on the basis of inconsistencies across repeated interviews. Indeed, a number of features of Granhag and Strömwall’s experimental setup facilitated the liars’ use of the *repeat* strategy (see Fisher et al., 2013). If these favorable conditions are removed, then contradictions may arise. Specifically, in the studies by Granhag and Strömwall the questions were about central aspects of the event and hence could be anticipated by liars (Fisher et al., 2013). Also, the participants knew they would be interviewed several times (Strömwall and Granhag, 2005), so they might have rehearsed their stories during the time between one interview and the next. In some studies (Granhag and Strömwall, 2000, 2001, 2002), the first interview was conducted shortly after the event, the second interview 4 days later, and the final interview 1 week after the second one. Arguably, the liars’ memory trace for their fabrications might still have been strong just 4 days after the event, and recalling the false story at this point might have prevented further memory decay (see Ebbesen and Rienick, 1998). Finally, Granhag and Strömwall did not use any manipulation to make it difficult for liars to fabricate a false story during the first interview, to encode that story, and to retrieve it during subsequent interviews. As explained below, all of these issues were addressed in the current research.

### Cognitive Load and Deception

Some of the new interviewing approaches to detect deception are based on the notion that, in interview settings, lying is cognitively more demanding than truth telling. Inventing a story is cognitively more difficult than just describing the truth, the lie must be plausible and consistent with everything the target knows or may learn, liars must remember their fabrications to provide consistent statements in the future, must monitor their online behavior not to look deceptive, as well as the targets’ reactions to make sure targets do not suspect deception, and must inhibit the truth, avoiding making slips of the tongue (Vrij et al., 2010a). All of these tasks consume cognitive resources. Cognitive psychologists have reasoned that lying requires access to executive control processes involved in suppressing the truth, searching for information in long term memory, and assembling a lie in working memory (see Gombos, 2006; Walczyk et al., 2013, 2014; Sporer, 2016). Supporting these notions, neuroimaging studies have shown that brain areas involved in working memory, response monitoring and conflict, inhibition, and multitasking are active during deception (see meta-analyses by Christ et al., 2009; Farah et al., 2014; Gamer, 2014; Lisofsky et al., 2014), and cognitive psychology studies have shown that lying requires greater access to executive control processes than truth telling (e.g., Debey et al., 2012; Visu-Petra et al., 2013; Fenn et al., 2015). Finally, some linguistic markers of cognitive load are present more often in deceptive than in truthful accounts (Hauch et al., 2015).

Because lying during an interview is cognitively more demanding than truth telling, artificially increasing the interviewees’ cognitive load further during questioning should be more detrimental for liars—who would then show observable signs of cognitive overload—than for truth tellers. One way of increasing cognitive load is asking interviewees to tell the story in the reverse (rather than the chronological) order (Vrij et al., 2008). Research shows that this procedure increases deception cues and observers’ accuracy in judging veracity (Vrij et al., 2008, 2012; Evans et al., 2013). Other ways of increasing cognitive load are asking participants to report the events in their non-native language (Evans et al., 2013) or to stare at the interviewer’s eyes (Vrij et al., 2010b), as well as to deplete the interviewees’ cognitive resources before the interview (Blandón-Gitlin et al., 2013). All of these strategies have been successful in creating behavioral differences between liars and truth tellers. Walczyk et al. (2005, 2009, 2012) introduced a more articulated interview approach called TRI-Con (Time Restricted Integrity-Confirmation) that involves posing questions to induce cognitive load during lying but facilitate responding during truth-telling. TRI -Con research has also yielded encouraging results (Walczyk et al., 2005, 2009, 2012). Based on this research, we also artificially increased the interviewees’ cognitive load to magnify the differences between liars and truth tellers.

### The Present Procedure

The goal of the current study was to design a new, cognitive-load based interview procedure to detect deception on the basis of inconsistencies across repeated interviews. Guilty participants committed a mock crime, whereas innocent participants performed several tasks at the request of an experimenter. All participants were then informed that they were suspects of the crime and would be interviewed. They had to convince the interviewer that they had performed the tasks of the innocent participants rather than committing the crime. Prior to the interview, guilty participants were invited to request from the experimenter as much information about the tasks carried out by the innocent participants as they deemed necessary to convince the interviewer that they were innocent. The interview was conducted almost immediately. One week later, the participants were *unexpectedly* interviewed again. Before each interview, participants were motivated to be convincing—those judged to be deceptive by the interviewer would be asked to write a text about a negative topic. Both interviews were identical and had the following specific features:

(a) *The focus of the interviews was solely on the alibi* (i.e., the innocent tasks), *not on the crime*. In certain circumstances, a strong focus on the alibi might be beneficial to ascertain the truth. To know whether a suspect is truthful or deceptive, that person’s statement may be checked against known facts. These known facts may concern the crime, or they may concern the alibi. Because offenders often take precautions not to be discovered, the police have a limited amount of information about the crime. However, under certain circumstances, the police can have information about the alibi. Good police work involves thoroughly investigating alibis, whether it is people, location or events. The suspects’ responses may then be compared against that information. For instance, imagine that a crime has been committed and suspect A tells the police that she had been watching TV Channel 1 at the time of the crime. The police may learn about the details of the program being shown at the time and question the suspect. If she did not watch the program, she might not be able to reply to a number of questions. If she asked someone else about the program contents, she might have asked only about the central details; so, including questions about some peripheral details may possibly reveal her lies. Suspect B tells the detective that, at the time of the crime, he accompanied a friend who lives in a distant small town to a nearby police station where the friend filed a complaint. The detective can contact the police station in the small town to get an accurate description of the place, as well as details specific of the time the suspect had purportedly visited the police station (who was at the station, what was happening, etc.).^1^ The current procedure could potentially be utilized in cases of this kind.
(b) *Both central and peripheral questions were asked*. Central questions inquired about the actions performed by the suspect and the details on which the suspect focused his or her attention. These central details cannot be changed without changing the story. Peripheral questions were about details and actions irrelevant for the story. These peripheral aspects can be changed with the main storyline remaining the same. These definitions were made after examining a large number of articles to ascertain how memory researchers had defined or understood central and peripheral details (e.g., Heuer and Reisberg, 1990; Burke et al., 1992; Ibabe, 2000; Ibabe and Sporer, 2004; Luna and Migueles, 2009). We anticipated that guilty suspects would request from the experimenter less information about peripheral than about central details, and hence liars would have little peripheral information to respond. Conversely, innocent suspects actually experienced the event and therefore would have more peripheral information than guilty suspects.
Even though our focus was on peripheral vs. central questions, there is an overlap with unexpected vs. expected questions (see Vrij et al., 2009; Lancaster et al., 2013). Certainly, although not all unexpected questions are peripheral, probably most peripheral questions are unexpected. Earlier we referred to the study by Vrij et al. (2009) exploring the potential of unexpected questions to identify pairs of liars. More recently, unexpected questions have been tested as a way to identify individual liars: because liars prepare the answers to expected (but not to unexpected) questions, they provide more details in responding to expected than to unexpected questions (Lancaster et al., 2013). Despite the parallelism, the present study differs from previous research in a number of ways: first, we examined the impact of peripheral and central questions on alibi construction; second, we used novel procedures (repeated interviews); and third, we examined dependent variables not explored in previous unexpected questions research—namely, inconsistencies and evasive answers.
(c) *The questions focused on very specific details* (e.g., the color of the office door). Most questions could be answered with just one word or a few words, and involved just one unit of information. This was done to facilitate measurement and coding of the dependent variables.
(d) *Interviewees were requested to respond as soon as possible after listening to each question*. This request, which is a component of another new interview technique to detect deception (Walczyk et al., ’s 2005, 2012; TRI-Con) and is also employed in response latency based approaches to detect guilty knowledge (Verschuere and De Houwer, 2011), was employed in the present study to increase cognitive load to magnify differences between liars and truth tellers. Asking to reply quickly is cognitively demanding: first, searching for information in long term memory requires time, particularly if the memory is poorly encoded. Second, if the requested information cannot be retrieved from memory, then the [deceptive] individual needs to invent a story, which is even more difficult (Vrij et al., 2010a), requiring more time. Having suspects perform these cognitively complex tasks (which require time and concentration) quickly requires mental effort, particularly for liars, whose memory trace for the event is weak or non-existent.
(e) A crucial feature was that *the interview was repeated after one week, and the participants were unaware that they would be interviewed again*.

### Hypotheses and Rationale

#### Request of Information to Prepare an Alibi

Guilty participants were invited to request information about the innocent tasks from the experimenter in order to be convincing during the interview. We assumed that guilty participants would not expect peripheral questions to be asked and would thus believe that a schematic linear account (what they did, where, and how) would suffice to be convincing. Therefore, we predicted that to prepare their alibi, guilty participants would request central rather than peripheral information from the experimenter (*Hypothesis 1*).

This hypothesis was in line with some considerations of the credibility assessment literature (e.g., Koehnken, 1996, 2004; Sporer, 2004, 2016; Volbert and Steller, 2014). Criteria-based Content Analysis (CBCA) is a set of 19 verbal criteria that are assumed to be more strongly present in truthful than in deceptive accounts (see Steller and Koehnken, 1989; Koehnken, 2004; Vrij, 2008). Scholars have suggested that several of the CBCA criteria reflect the notion that truth tellers, who describe an episodic autobiographical event, will spontaneously include in their statements more spatial, temporal, and self-related information than liars (Sporer, 2004), as well as more script-deviant details (i.e., information that does not fit the mental schema or “script” that people may have of a specific kind of event; see Schank and Abelson, 1977). On the contrary, liars “will not come up with the idea of integrating such information” (Volbert and Steller, 2014, p. 212; see also Sporer and Kuepper, 1995; Koehnken, 1996, 2004; Sporer, 2016). Liars may not think specific, contextual, or script-deviant details to be important to fabricate a convincing account. Examining this issue in the context of alibi construction is a novel enterprise, and it can suggest new ways of taking advantage of the weaknesses of alibi fabrication to ascertain the truth.

#### Inaccurate Answers as a Deception Cue

Innocent participants performed the innocent tasks. Therefore, they probably encoded relevant information and would be able to retrieve that information from memory during both interviews (though some memory decay was expected). This would result in high memory accuracy, as well as little inconsistencies across interviews for truth tellers. Conversely, guilty participants did not perform the innocent tasks. All they knew about these tasks was what they had learned from the experimenter, who simply answered their questions. This information may be incomplete and poorly encoded. Because of this, we predicted that in both interviews guilty participants would correctly answer fewer questions than innocent participants (*Hypothesis 2*). Furthermore, this effect would be stronger when answering peripheral rather than central questions (*Hypothesis 3*) because, according to Hypothesis 1, guilty participants would have some information about central details but no information about peripheral details.

#### Inconsistencies

We compared the suspects’ answers to each individual question across interviews. If the two answers were semantically the same, this was coded as consistent. If they were different, this was coded as inconsistent. We predicted that guilty participants would change their answers from one interview to the next (i.e., would show inconsistencies) more often than innocent participants (*Hypothesis 4*), and that this effect would be stronger for peripheral than for central details (*Hypothesis 5*). Unlike Granhag and Strömwall (1999, 2000, 2001, 2002; Granhag et al., 2003; Strömwall and Granhag, 2005), we created the conditions for inconsistencies to appear among liars by impairing a *repeat* strategy. First, although liars may have anticipated being requested to provide a full narrative, they were asked very specific questions. Second, some of these specific questions were about peripheral details that liars may not have thought of in preparing their alibi. Third, the retention interval was relatively long (1 week). Fourth, the participants were unaware that they would be interviewed again; this made rehearsal less likely during the retention interval and hence facilitated memory decay, particularly for poorly encoded memories—that is, those of liars.

Concerning peripheral details, because liars would have less peripheral information, they would have to invent the answers during the first interview and recall those answers (provided they wanted to be consistent) during the second interview. However, the cognitive load induction procedure was designed to undermine these strategies. Both encoding and retrieving information from memory consume cognitive resources and require attention and concentration. Research has consistently shown that a secondary task that consumes cognitive and attentional resources hinders the *encoding* of new information, and increases reaction times during *retrieval* (e.g., Baddeley et al., 1984; Craik et al., 1996; Iidaka et al., 2000). Similarly, Chandler and Sweller (1996) demonstrated that cognitive load has a detrimental effect on learning. Therefore, we expected that during the first interview the induced cognitive load would hinder the encoding of the deceptive answers hastily made up on the spot. Cognitive load could also have a negative effect on the retrieval of these answers during the second interview, because the participants had been asked to reply quickly.

Cognitive load would presumably affect not only the liars’ answers to questions about peripheral details, but also their answers to questions about central details. Indeed, cognitive load may increase the difficulty in retrieving poorly encoded or incomplete memories about central details during both interviews, also contributing to generating inconsistencies for central details in liars (as predicted in Hypothesis 4).

#### Evasive Answers

We defined evasive answers as replies that contain no relevant information. For example, statements such as “I don’t know,” “I don’t remember” or the like qualify as evasive answers. Replying “there was no poster” when asked on which wall there was a poster (provided there was actually a poster) should also be coded as an evasive answer—the suspect gives a response but the response contains no substantive answer to the question. Guilty participants asked to reply quickly and unable to retrieve the requested information from memory or to invent a plausible lie may ultimately resort to providing evasive answers to get out of the stalemate. They have been asked to reply quickly but they cannot find the relevant information and cannot quickly invent a lie, so they may see saying “I don’t know” as the only way to escape from the situation. Thus, we predicted that guilty suspects would provide more evasive answers than innocent suspects (*Hypothesis 6*), particularly in response to peripheral questions (*Hypothesis 7*).

#### Predictive Value of the Dependent Variables on Veracity

In addition to examining the impact of guilt status on response accuracy, consistency, and evasive answers, we also ran several binary logistic regression analyses to examine whether guilty and innocent participants could be correctly identified on the basis of each of these variables. These analyses were run both with no cross-validation and with the leave-one-out cross-validation method (Note: in only one case did classification rates vary as a function of whether cross-validation was used; see the results section). We predicted substantial classification rates would be attained.

## Materials and Methods

### Participants

Participants were 48 White undergraduate criminology students (30 females and 18 males; *M*_age_ = 19.71 years, *SD* = 2.70) who volunteered to take part in the study in exchange for an academic incentive. Guilty participants (*n* = 24) had their lectures in the morning whereas innocent participants (*n* = 24) had their lectures in the evening. In the criminology degree, students in each group (i.e., morning or evening group) hardly know or interact with those of the other group. Therefore, our guilty and innocent participants could hardly exchange any information about their respective tasks. Group allocation is made by the Law School administration, and is based on the first letter of each student’s family name. Guilty and innocent participants did not differ significantly in terms of gender (15 females and 9 males in each condition) or age (guilty participants: *M*_age_ = 19.42 years, *SD* = 1.82; innocent participants: *M*_age_ = 20.00 years, *SD* = 3.38; *t*(46) = 0.75, *p* = 0.460.

### Procedure

The experimental sessions were conducted over several weeks. During Week 1, guilty participants committed the mock crime and were interviewed for the first time. During Week 2, they were interviewed for the second time. Similarly, during Week 3, innocent participants performed the innocent tasks and were interviewed. Then, during Week 4, innocent participants were interviewed again.^2^ All instructions, experimental procedures, and interviews were scripted. Scripts (in Spanish) are available from the first author on request. The procedures were in accordance with institutional, national, and international (the American Psychological Association’s) ethics guidelines. Informed consent was obtained from all participants, and participants were free to leave the experiment at any point.

#### Phase 1: Guilty and Innocent Participants’ Tasks

Each participant individually met an experimenter at a specific location in the hall of the School of Psychology.

##### Guilty participants

The experimenter escorted each guilty participant to a laboratory room. After signing an informed consent form, the participant received the door key of Seminar Room 126 from the experimenter. The experimenter asked the participant to imagine that s/he simply happened to find the key. The participant’s task was to use the key to slip into the seminar room, steal a wallet (with some money inside) that was on top of a table, and return.

When the participant had returned with the wallet, the experimenter turned on a video camera and told the participant that s/he (the participant) was suspected of having stolen the wallet and was about to be interviewed. The experimenter explained that other participants had not stolen the wallet but, instead, had performed several tasks with an experimenter in an office room. The participants’ goal was to convince the interviewer that s/he was innocent and had been doing the alternative tasks. If s/he was successful in convincing the interviewer, then s/he would not be asked to do anything else and would leave straightaway. However, if the interviewer judged the participant as being deceptive, then s/he would be asked to write a text at least one page long about her/his least favorite high school course. At this point, the following instructions were delivered:

*Imagine this situation is real. Imagine that to avoid being sent to jail you have to convince the police you were not involved in the crime. To have an alibi to convince the interviewer that you are innocent, please ask me everything you want to know about what the innocent participants did*.

The participant asked the experimenter questions about the activities carried out by the innocent participants. The experimenter replied to all of the questions providing only the specific information requested by the participant, and nothing more. This conversation was entirely videotaped. After the participant finished asking the questions, the experimenter left the room and the interviewer stepped in.

##### Innocent participants

The experimenter escorted each innocent participant to an office room. The room had been set for the experiment such that there were specific peripheral details that could be used to question the participants about (a poster on a wall depicting Homer Simpson, a vase with yellow flowers on a shelf, etc.). After signing an informed consent form, the participant was told that s/he had to perform four different tasks and could spend up to 4 min on each. The first task was playing Tetris on a computer, the second task was performing a number of specific arithmetic operations on a sheet of paper, the third task was searching for the definition of “dinosaur” in Wikipedia and pasting it in a Microsoft Word file, and the final task consisted of watching a brief (less than 2 min.) fragment of a documentary and typing the answers to some questions about the documentary in a Microsoft Word file.

After the tasks, participants were told that some other participants had not performed these activities but, instead, had stolen a wallet from a seminar room. The experimenter also told the innocent participant that s/he (the participant) was a suspect of the theft, was about to be interviewed, and her/his goal was to convince the interviewer that s/he was innocent of the theft and had been doing the alternative tasks. The rest of the instructions were identical to those given to the guilty participants, except that innocent participants were not invited to ask the experimenter about the innocent tasks.

Next, the experimenter escorted the innocent participant to a laboratory room to be interviewed. Guilty and innocent participants were interviewed in the same room, and the setup of the room, including the specific location of the camera and chairs, was identical across all interviews.

#### Phase 2: The Interviews

All participants were interviewed twice—first shortly after performing the guilty or innocent tasks, and then again 1 week later. The interviews were run by 22 psychology undergraduate students (all females, *M*_age_ = 22.00 years, *SD* = 1.16) who participated in exchange for an academic incentive. Each interviewer conducted 4.36 interviews on average (because of unforeseen circumstances, not all interviewers conducted the same number of interviews).^3^ The interviewers running Interview 1 (*n* = 11) were different from those running Interview 2 (*n* = 11). Each interviewer questioned only guilty or only innocent participants. Before running the interview, interviewers were given detailed written and oral instructions, as well as a written script with all of the questions. All interviewers were blind with respect to the guilty or innocent status of the participants, the details of the alibi, and the purposes of the experiment.

At the beginning of the interview, the interviewer told the participant:

*As you know, someone stole a wallet from Seminar Room 126 and you are a suspect in this theft. I am going to ask you a number of questions about your whereabouts during the last half an hour or so. You have to reply and, in order not to appear suspicious, you better do not allow much time to pass between my question and your answer. I am going to record how long it takes*.

Then, the interviewer set a timer and began the interview. All questions are displayed in Appendix 1 (in supplementary online materials). All participants were expected to (and did) reply “no” to the first question and “yes” to the second question. These two questions were not scored. Questions 3 through 18 were eight central (i.e., these questions requested central information) and eight peripheral (i.e., the question asked for peripheral details) questions selected on the basis of a preliminary study (Appendix 2 in supplementary online materials). The interview was video recorded. The interviewer was out of the frame, sitting in front of the suspect. The camera was facing the suspect, who was sat on a chair and was visible from the ankles up.

After the interview was over, the interviewer brought in the experimenter, who had been waiting in an adjacent small room. The interviewer indicated in a form whether she thought the suspect was lying or telling the truth, as well as her confidence in the judgment on a 1 (*little*) to 5 (*much*) scale. Then she silently showed her responses to the experimenter and left. At this point, the experimenter (a) thanked the participant for his/her participation, (b) reminded the participant about the need not to disclose information about the experiment to her/his peers, and (c) reminded the participant that s/he had to come back the following week to take part in a separate experiment. The experimenter also escorted suspects judged (by the interviewer) to be deceptive to the library, where they wrote a text about their least favorite course in high school. They were requested to place this text into the first author’s mailbox before leaving. All of the suspects required to write the text did so.

The second interview took place 7 days later. The instructions were almost identical to those of the first interview, except that before the second interview neither guilty nor innocent participants were invited to ask the experimenter about the innocent tasks. The interview procedure, questions, and question order were the same as in Interview 1. After each individual interview, interviewers made a veracity judgment and rated their confidence. The experimenter explained to the participants why it was important not to tell the other participants that they had been interviewed again until all the experimental sessions were over, and asked them to sign a form promising not to disclose this information until data collection was complete.

#### Post-experiment Questionnaire (Manipulation Checks)

Next, the experimenter had the participant complete a post-experiment questionnaire. Most of the questions were asked twice, once referring to “last week” and once referring to “today.” Most questions had to be answered on a 1 (low on the dimension) to 5 (high on the dimension) scale. The following was in the questionnaire:

To assess veracity manipulation: *To what extent were your answers during last week’s/today’s interview deceptive?, To what extent were your answers during last week’s/today’s interview truthful?* (reverse scored), *Did you try to lie during last week’s/today’s interview?*
To assess participants’ event knowledge: *How much information did you have at the beginning of last week’s interview about the tasks performed in the office room with an experimenter*?, *How much of this information did you remember today?*
To assess participants’ experience of cognitive load: *How much mental effort did you exert when replying to the questions during last week’s/today’s interview?, How easy or difficult was last week’s/today’s interview for you?, How difficult was it for you to be convincing during last week’s/today’s interview?*
To assess awareness of experimental manipulations: *Had you anticipated you would be interviewed again this week about the same issue?* (No/Yes), *Did anyone tell you before today anything about what would happen during the second week?* (No/Yes).

In addition to completing the questionnaire, participants judged to be deceptive by the interviewer during the second interview were also requested to write about their least favorite high school course. Before leaving, participants left the completed questionnaire (and the high school text if they wrote one) in the first author’s mailbox. Participants were debriefed later in class.

### Transcribing and Coding

#### Guilty Participants’ Alibi Preparation

Guilty suspects asked the experimenter about the innocent tasks. These conversations were videotaped, the verbal content was subsequently transcribed, and the accuracy of the transcripts was checked against the original videotape by a research assistant. Two coders were given the definitions and examples of central and peripheral questions and independently coded all questions as either central, peripheral, or irrelevant (i.e., not related to the innocent activities). Out of 210 questions coded^4^, 187 (89%) were classified as central by both coders. The number of questions coded as peripheral was only one according to Coder 1 and four according to Coder 2. Across all three kinds of questions, percent agreement was 93.3%. Disagreements were resolved by discussion. The same two coders also independently assessed on a 0 (*no more information than requested*) to 5 (*much more information than requested*) scale whether the experimenter provided more information than requested in answering each of the questions by all guilty suspects. Virtually all answers were rated 0 by both coders.

#### Accuracy and Consistency Responses

Two additional coders received all the videotaped interviews, detailed coding instructions (both in written and orally) and an Excel spreadsheet.^5^ The coders had to transcribe and code each suspect’s answers in the spreadsheet. Suspects were presented in a random order so that not all guilty or all innocent suspects were coded first. Specifically, the two coders were requested to independently (a) view the videos and record on the Excel sheet each suspect’s reply to each question during the first and second interview; (b) compare the two answers given by each suspect to each question and code whether the answers were the same (1) or different (0) (*consistency codings*); and (c) for each individual answer, code whether it was correct (1) or incorrect (0) (*response accuracy codings*). Note, the coders were informed about correct responses just before doing this latter task. The two coders independently coded data for all 96 interviews. Reliability ranged from substantial to almost perfect (Landis and Koch, 1977; Vieira and Garrett, 2005): for consistency, percent agreement = 89.55%, and *Kappa* = 0.72; for response accuracy in Interview 1, percent agreement = 93.04%, and *Kappa* = 0.86; for response accuracy in Interview 2, percent agreement = 92.27%, and *Kappa* = 0.76. Disagreements were resolved by discussion. All transcripts and coding decisions were subsequently checked by a researcher.

#### Evasive Responses

Two additional coders received written definitions and examples of evasive answers, as well as the 96 interview transcripts. They had to code whether each individual answer was evasive or not. For Interview 1, percent agreement was 98.44% and *Kappa* was 0.94; for Interview 2, percent agreement was 98.56% and *Kappa* was 0.95. Disagreements were resolved by discussion.

## Results

### Manipulation Checks

#### Amount of Information Obtained by Guilty Suspects

We had to ensure that the guilty participants did not receive more information than requested from the experimenter. The unrequested information could allow guilty suspects to answer questions they did not anticipate, that is, questions about issues they did not deem relevant. Coders rated on a 0-to-5 scale the extent to which the experimenter over-informed guilty participants. Out of 215 questions asked by all 24 guilty suspects, 210 got a score of 0 and five a score of 1 (*M* = 0.02, *SD* = 0.15). In short, guilty suspects did not obtain more information than requested from the experimenter.

#### Post-experiment Questionnaire

The scores for separate questions tapping the same construct in the post-experimental survey were combined (see Cronbach’s Alphas in **Table 1**) and analyses were run comparing guilty and innocent suspects. As expected, across both interviews guilty participants reported being more deceptive, having less information, and experiencing more cognitive load than innocent participants (**Table 1**). These findings show that our manipulations were successful. None of the participants indicated that someone else told them what would happen in the second week, but four truth tellers and five liars had contemplated the possibility of being interviewed again.

**Table 1:** Manipulation Checks.

	Guilty suspects		Innocent suspects		*t*(46)	*p*	*d*	95% CI	Number of questions	Alpha
	*M*	*SD*	*M*	*SD*						
***Deceptiveness***										
Interview 1	3.83	0.86	1.31	0.44	12.80	<0.001	3.69	[2.76,4.62]	3	0.93
Interview 2	3.92	0.87	1.47	0.50	11.94	<0.001	3.45	[2.56,4.35]	3	0.91
***Amount of information***										
Interview 1	3.00	1.22	4.17	1.09	-3.50	0.001	-1.01	[-1.61,-0.41]	1	-
Interview 2	2.83	1.24	3.63	1.06	-2.38	0.021	-0.69	[-1.28,-0.11]	1	-
***Cognitive load***										
Interview 1	2.99	0.96	1.97	0.86	3.86	<0.001	1.12	[0.51,1.73]	3	0.76
Interview 2	3.18	0.76	2.33	0.89	3.54	<0.001	1.03	[0.43,1.63]	3	0.75

*>All variables were measured on 0-to-5 scales. Three Guilt Status (Guilty vs. Innocent participant) × Interview (Interview 1 vs. Interview 2) Analyses of Variance (ANOVAs) were also run (available from the first author on request). The main effect of guilt status was significant in all three ANOVAs, and neither the interview main effect nor the Guilt Status × Interview interaction were significant in either ANOVA.*

### Request of Information to Prepare an Alibi

Hypothesis 1 predicted that, to prepare their alibi, guilty participants would request central rather than peripheral information from the experimenter. The data strongly supported this hypothesis. In all, the 24 guilty participants asked 215 questions. Of these, 197 (91.63%) were about central information, and only five (2.33%) were about peripheral information. The remaining 13 questions (6.05%) were irrelevant questions (i.e., questions unrelated to the innocent activities).^6^

### Inaccurate Answers as a Deception Cue

A 2 (Guilt Status: Guilty vs. Innocent) × 2 (Question Type: Central vs. Peripheral Questions) × 2 (Interview 1 vs. Interview 2) mixed Analysis of Variance (ANOVA), with repeated measures on the latter two variables, was run on response accuracy (i.e., proportion of accurate answers). Accuracy was higher in replying to central, *M* = 0.70, *SD* = 0.30, than to peripheral questions, *M* = 0.47, *SD* = 0.31, *F*(1,46) = 58.02, *p* < 0.001, $\eta_{p}^{2}$ = 0.558, 90% CI [0.385,0.661]. Furthermore, as predicted by Hypothesis 2, guilty participants, *M* = 0.33, *SD* = 0.13, gave considerably fewer accurate answers than innocent participants, *M* = 0.84, *SD* = 0.09. *F*(1,46) = 270.18, *p* < 0.001, $\eta_{p}^{2}$ = 0.855, 90% CI [0.782,0.889]. However, contrary to Hypothesis 3, the Guilt Status × Question Type interaction was not significant, *F*(1,46) = 0.36, *p* = 0.551, $\eta_{p}^{2}$ = 0.008, 90% CI [0.000,0.093]. Unexpectedly, the Question Type × Interview interaction was significant, *F*(1,46) = 7.70, *p* = 0.008, $\eta_{p}^{2}$ = 0.143, 90% CI [0.022,0.294].^7^

To examine whether guilty and innocent participants could be correctly identified on the basis of accuracy scores, we ran a binary logistic regression analysis with accuracy scores as the predictor variable. As shown in **Table 2** (first row), classification accuracy was perfect, as was the model fit to the data.

**Table 2:** Classification rates of binary logistic regression analyses, and interviewers’ accuracy rates (Bottom Row).

Predictors	Classification Rates						
	Truths (%)	Lies (%)	Overall (%)	Model χ*^2^*	*df*	*p*	Nagelkerke’s *R*^2^
Response accuracy	100.00	100.00	100.00	66.54	1	<0.001	1.00
Consistency	70.83	66.67	68.75	15.88	1	<0.001	0.38
Consistency for central questions^a^	87.50	58.33	72.92	22.20	1	<0.001	0.49
Consistency for peripheral questions							
Evasive answers	87.50	87.50	87.50	35.09	1	<0.001	0.69
Evasive answers to central questions^b^	87.50	83.33	85.42	39.07	2	<0.001	0.74
Evasive answers to peripheral questions							
Consistency (recoded)^c^	70.83	62.50	66.67	18.15	1	<0.001	0.42
Consistency (recoded) for central quests^a^	87.50	62.50	75.00	22.19	1	<0.001	0.49
Consistency (recoded) for peripheral qs.							
Combined variable^d^	95.83	91.67	93.75	54.13	1	<0.001	0.90
Interviewers’ accuracy rates	70.70	39.67	53.77	-	-	-	-

*^a^Only predictor retained in binary logistic regression analysis with the forward - likelihood ratio method.*

*^b^With the leave-one-out cross-validation method, the classification rate for lies was slightly lower (79.17%), and the overall classification rate was 83.33%.*

*^c^Consistency excluding all consistency instances caused by evasive answers.*

*^d^Inconsistent answers and consistent answers due to repeated evasive answers coded as 0, and all other consistent answers coded as 1.*

### Consistency

A Guilt Status (Guilty vs. Innocent) × Question Type (Central vs. Peripheral Questions) mixed ANOVA on consistency scores (proportion of consistent answers across interviews) yielded a significant guilt status main effect, *F*(1,46) = 17.08, *p* < 0.001, $\eta_{p}^{2}$ = 0.271, 90% CI [0.101,0.421]. Supporting Hypothesis 4, innocent suspects, *M* = 0.89, *SD* = 0.09, were more consistent than guilty suspects, *M* = 0.71, *SD* = 0.20. The interaction was significant, *F*(1,46) = 8.84, *p* = 0.005, $\eta_{p}^{2}$ = 0.161, 90% CI [0.031,0.313], and revealed that the difference in terms of consistency between innocent and guilty suspects was larger in responding to central questions, *M*_innocent_ = 0.93, *SD* = 0.10, *M*_guilty_ = 0.67, *SD* = 0.24, *p* < 0.001, *d* = 1.41, 95% CI [0.78,2.05], than in responding to peripheral questions, *M*_innocent_ = 0.86, *SD* = 0.13, *M*_guilty_ = 0.76, *SD* = 0.22, *p* = 0.056, *d* = 0.55, 95% CI [-0.02,1.13]. This latter finding was opposite to Hypothesis 5. The question type main effect was not significant, *F*(1,46) < 1.

Based on the guilt status main effect in the ANOVA, we ran a binary logistic regression analysis with consistency scores across central and peripheral questions as the only predictor variable. As shown in **Table 2** (second row), 70.83% of truth tellers and 66.67% of liars were classified correctly (overall classification rate was 68.75%). Also, because the Guilt Status × Question Type interaction was significant in the ANOVA, we ran an additional binary logistic regression analysis with the forward - likelihood ratio (LR) method with two predictors: consistency in replying to central questions and consistency in replying to peripheral questions. Only consistency for central questions was retained, and the classification rates were 87.50% for truth tellers and 58.33% for liars (72.92% overall classification rate; see **Table 2**).

### Evasive Answers

A Guilt Status (Guilty vs. Innocent) × Question Type (Central vs. Peripheral Questions) × Interview (1 vs. 2) ANOVA revealed that the proportion of evasive answers was higher in responding to peripheral, *M* = 0.25, *SD* = 0.21, than to central questions, *M* = 0.09, *SD* = 0.15, *F*(1,46) = 29.29, *p* < 0.001, $\eta_{p}^{2}$ = 0.389, 90% CI [0.203,0.524]. It was also higher among guilty, *M* = 0.28, *SD* = 0.13, than among innocent suspects, *M* = 0.06, *SD* = 0.07, *F*(1,46) = 52.54, *p* < 0.001, $\eta_{p}^{2}$ = 0.533, 90% CI [0.357,0.642]. This result supported Hypothesis 6. The Guilt Status × Question Type interaction predicted in Hypothesis 7 was also significant, *F*(1,46) = 4.37, *p* = 0.042, $\eta_{p}^{2}$ = 0.087, 90% CI [0.002,0.228]. Even though guilty participants provided significantly more evasive answers than innocent participants in responding both to central, *M*_guilty_ = 0.17, *SD* = 0.19, *M*_innocent_ = 0.01, *SD* = 0.02, *F*(1,46) = 16.68, *p* < 0.001, $\eta_{p}^{2}$ = 0.266, 90% CI [0.097,0.416], and to peripheral questions, *M*_guilty_ = 0.39, *SD* = 0.18, *M*_innocent_ = 0.11, *SD* = 0.14, *F*(1,46) = 38.15, *p* < 0.001, $\eta_{p}^{2}$ = 0.453, 90% CI [0.268,0.578], the effect was larger for peripheral than for central questions.

A binary logistic regression analysis with evasive answers across central and peripheral questions as the predictor variable yielded classification rates of 87.50% both for truth tellers and liars (**Table 2**). Similar classification rates were obtained when evasive answers for central and peripheral questions were entered as two separate predictors in a logistic regression analysis with the forward - LR method; these classification rates were slightly lower but still around the 80% rate when cross validation was used (see **Table 2**).

### Additional Consistency Analyses

The above analyses revealed that (a) *consistency* is a valid indicator of *truth*, and (b) *evasive answers* are a valid indicator of *lies*. Recall that if a participant gave the same answer to the same question in both interviews, that was coded as a consistent answer ( = a truth indicator). However, if the same answer given to both interviews was an evasive answer ( = a lie indicator), this was also coded as consistent ( = truth indicator). This suggests that had we dismissed evasive answers in coding consistency, the discrimination power of consistency ratings would have been even higher.

We addressed this issue in two different ways. First, we ran all consistency analyses again excluding all instances of consistency that were caused by evasive answers from these analyses (these cells were left empty in the SPSS dataset). The findings (available from the first author on request) mirrored those of the above consistency analyses. Logistic regression analyses with the recoded consistency scores also yielded classification rates similar to those obtained with the original consistency scores (see **Table 2**).

Second, we created a new variable combining consistency and non-evasive answers. As usual, inconsistent answers were coded as 0 and consistent answers were coded as 1. However, if consistency was due to evasive answers, this was coded as 0. In this way, values of 1 denoted truthfulness and values of 0 denoted deception. We expected truth tellers to score significantly higher than liars on this new variable.

**FIGURE 1:**
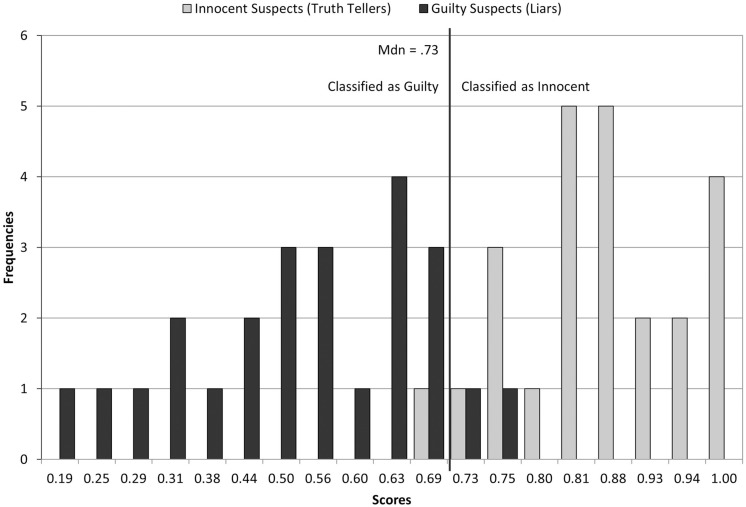
**Distribution of guilty and innocent suspects’ scores in the combined variable**.

A 2 (Guilt Status) × 2 (Question Type) mixed ANOVA revealed that scores on the new combined variable were significantly lower for peripheral, *M* = 0.62, *SD* = 0.24, than for central questions, *M* = 0.76, *SD* = 0.26, *F*(1,46) = 16.34, *p* < 0.001, $\eta_{p}^{2}$ = 0.262, 90% CI [0.095,0.413]. Also, as predicted, innocent suspects scored significantly higher, *M* = 0.86, *SD* = 0.09, than guilty suspects, *M* = 0.52, *SD* = 0.16, *F*(1,46) = 79.64, *p* < 0.001, $\eta_{p}^{2}$ = 0.634, 90% CI [0.479,0.721]. Interestingly, the Guilt Status × Question Type interaction was not significant, *F*(1,46) < 1, indicating that the difference between innocent and guilty suspects was the same for central as for peripheral questions. A binary logistic regression analysis with the combined variable as the sole predictor attained a 95.83% classification rate for truth tellers (23 out of 24 were classified correctly), and a 91.67% classification rate for liars (22 out of 24 liars were classified correctly), with an overall classification rate of 93.75%. As shown in **Figure 1**, the overlap between the distribution of guilty and innocent suspects was very small; no guilty suspect scored above 0.75 and no innocent suspect scored below 0.69.

### Interviewers’ Accuracy

Each interviewer conducted 4.36 interviews on average. The overall accuracy rate of each interviewer was calculated, and an ANOVA was run with interviewer (*N* = 22) as the unit of analysis. Guilt Status (Guilty vs. Innocent) and Interview (1 vs. 2) were entered as between-subject independent variables in the ANOVA, and the dependent variable was the interviewers’ accuracy. Only the main effect of guilt status was significant, *F*(1,18) = 9.59, *p* = 0.006, $\eta_{p}^{2}$ = 0.348, 90% CI [0.068,0.546]. Accuracy in detecting truths (i.e., in identifying innocent suspects), *M* = 70.70%, *SD* = 22.86, was higher than accuracy in detecting lies (identifying guilty suspects), *M* = 39.67%, *SD* = 21.94. This was due to a strong truth bias among interviewers: 63.91% of the interviewers’ judgments were truth judgments, and only 36.09% were lie judgments, *t*(21) = 2.89, *p* = 0.009, *d* = 1.23, 95% CI [0.32,2.15]. To facilitate comparison with the logistic regression classification rates, we included interviewers’ accuracy rates in **Table 2** (bottom row).

Additional Guilt Status × Interview ANOVAs were conducted on the interviewers’ age, number of interviews conducted, proportion of deception (vs. truth) judgments, and judgmental confidence. No main effect or interaction reached significance. Surprisingly, the correlation between interviewers’ accuracy and their confidence scores was substantial and significant, *r*(*N* = 22) = 0.47, *p* = 0.028.

## Discussion

Recent deception research has focused on designing interviewing approaches to increase behavioral differences between truth tellers and liars (Vrij et al., 2010a; Vrij and Granhag, 2012; Vrij, 2014). The current study is in line with this new research trend. In this paper, we described the first steps to create an interview procedure to detect deception in which the senders’ cognitive load is increased in order to elicit within-suspect inconsistencies and evasive answers in liars.

### Liars’ Alibi Preparation: Central Event Details Were Prominent

We predicted, and found, that guilty participants would request primarily central rather than peripheral information to prepare their alibis (Hypothesis 1). This finding is consistent with the credibility assessment literature, which shows that deceptive statements are more script-like, schematic, and devoid of details (sensory, perceptual, and contextual information) compared to truthful statements (e.g., Sporer, 2004, 2016; Masip et al., 2005). CBCA scholars have suggested that truth tellers spontaneously include more spatial, temporal, self-related, and script deviant information than liars, who may not think of including these kinds of details (Koehnken, 1996, 2004; Sporer, 2004, 2016; Volbert and Steller, 2014).

The present study adds to the scant alibi generation literature (Culhane et al., 2008; Allison et al., 2011; Olson and Charman, 2012), and the findings have implications for deceptive alibi detection. Although some research has been conducted on alibi believability (e.g., Olson and Wells, 2004; Allison et al., 2012), only rarely has deception research focused on alibies (see Culhane et al., 2013). In the current study, the suspects’ failure in obtaining sufficient [peripheral] information from an “informed witness” (the experimenter) resulted in inaccurate, evasive, and inconsistent answers during a subsequent investigative interview. Culhane et al. (2013, Study 2) found that participants did not perform better than chance in determining whether videotaped alibi statements were truthful or deceptive. The current experiment showed that, under the right circumstances and with strategic interviewing, certain verbal cues can expose deceptive alibies.

### Interviewees’ Response Accuracy: Liars Performed Poorly

The interview questions focused on the innocent activities. Since truth tellers performed these activities, we assumed truth tellers encoded rich episodic memories and would therefore be able to recall information more accurately than liars during the interviews (Hypothesis 2). The data supported this prediction. However, contrary to Hypothesis 3, this effect was not more pronounced in answering peripheral questions than central questions. This finding suggests that although liars requested and obtained central information from the experimenter, this information either (a) was not sufficient to answer all the central questions in the interview, or (b) was poorly encoded by liars, who could not retrieve it later during the interview. Indeed, manipulation checks showed that liars reported having less information before each interview than truth tellers (see **Table 1**).

Classification rates obtained with a binary logistic regression analysis with accuracy scores as the predictor variable were perfect. This finding is rather impressive and came as a surprise. The main focus of the present research was on inconsistencies, and although we did expect differences between truth tellers and liars in response accuracy, we expected these to occur mostly in responding to peripheral questions and to allow for only moderate discrimination. The present outcome suggests that whenever the police can gather sufficient accurate central and peripheral information about the alibi, they should ask suspects about the alibi.

### Catching Liars: Consistency and Evasive Answers Were Effective

Prior research examining consistency across repeated interviews has found no difference between truth tellers and liars, presumably because liars adopt a *repeat strategy*—that is, liars actively try not to contradict themselves across interviews (Granhag and Strömwall, 1999). In this study we conducted repeated interviews. However, we tried to impede the use of the repeat strategy by asking focused questions (some of which were about peripheral details), by requesting participants not to delay their answers (thus increasing their cognitive load), by having an expanded retention interval, and by keeping participants unaware they would be interviewed again. These conditions worked well, as we found liars’ responses to be less consistent (Hypothesis 4) and to contain more evasive answers (Hypothesis 6) than truth tellers’ responses. Also, as predicted (Hypothesis 7), the difference between liars and truth tellers in terms of evasive answers was larger for peripheral than for central questions. It is interesting, however, that the difference was statistically significant also for central questions.

Contrary to Hypothesis 5, inconsistencies in replying to peripheral questions did not differentiate between innocent and guilty suspects better than inconsistencies in replying to central questions. Examination of the means reveals that whereas innocent suspects were somewhat more consistent in replying to central than peripheral questions, guilty suspects were somewhat more consistent in replying to *peripheral* (0.76 on a 0-to-1 scale) than to central questions (0.66). This high degree of consistency among guilty suspects replying to peripheral questions decreased the difference between innocent and guilty suspects’ consistency for peripheral questions; as a consequence, Hypothesis 5 was not supported.

Why were liars’ answers to peripheral questions so consistent? Research on the *forced confabulation effect* has shown that when people self-generate answers to *unanswerable* questions (e.g., about non-existent details in a perceived event), they might incorporate their confabulated responses in their memory network and provide the same answer during subsequent interviews (e.g., Pezdek et al., 2007). In the current experiment, liars had little peripheral information; therefore, most peripheral questions were unanswerable for them. Having to think and generate an answer to unexpected questions could have made it possible to incorporate that information to their memory, information that was available at the second interview. An alternative explanation suggests that when guilty participants were asked peripheral questions and were unable to retrieve any response, they experienced the situation as unexpected, salient, and thrilling. This may have increased their attention, facilitating the memory encoding of the unexpected or salient situation (including the response given) during Interview 1, as well as memory retrieval during Interview 2 (for evidence on the positive effect of stimulus unexpectedness on memory, see Pezdek et al. (1989); for evidence showing that emotional events are better remembered than expected events, see Bradley et al. (1992)).

The predictive value of consistency scores and the proportion of evasive answers to classify suspects as truth tellers or liars—tested with logistic regression analyses—was around 70% for consistency, and above 85% for evasive answers (**Table 2**). When consistency and evasive answers were combined as described in the results section, 96% of truth tellers and 92% of liars were classified correctly. All of these classification rates were much better (and considerably less truth biased) than the interviewers’ accuracy rates. Overall interviewers’ accuracy was 54%, which is virtually identical to the meta-analytical mean reported by Bond and DePaulo (2006). Interviewers’ accuracy was substantially higher for truths (71%) than lies (40%), which is also typical in the deception literature (Levine et al., 1999; Bond and DePaulo, 2006; Street and Masip, 2015; Street and Richardson, 2015).

Of course the interviewers were at a disadvantage. Although they used an interview procedure strategically designed to induce greater differences between truth tellers and liars, they had no training in assessing the truth and deception cues relevant in this study. They also did not have a chance to evaluate interviewees both times. Nevertheless, the interviewers’ accuracy data are interesting for at least two reasons. First, because they provide a fair comparison condition to the logistic regression. Second, because they suggest that high accuracy in deception detection can be reached outside the immediate interview. When a strategic interview procedure is used, even if the interviewers themselves cannot unambiguously assess deception online, other trained individuals evaluating such interviews may reach high accuracy levels. Training in strategic interviewing and assessment of cues likely to be elicited in the interview would seem to be productive.

### Practical Implications, Caveats, and Limitations

The current procedure could add to the extant arsenal of strategic interviewing approaches to detect deception. It has a number of distinctive features: it focuses solely on the alibi, uses repeated interviews (specifically designed to create inconsistencies in liars but not in truth tellers), and strategically employs both questions about central details and questions about peripheral details. It shows that indicators of cognitive load other than those examined in previous research can be useful to sort liars from truth tellers. An interesting contribution (from a research perspective, but which may also impact practice) is that, contrary to the current view in the field, inconsistencies across repeated interviews *can* reveal deception if certain measures are taken. An additional advantage of this interview technique is its brevity. But its most outstanding feature is its extremely high classification rate, outperforming the extant cognitive-load based approaches.

However, more research is needed before using the current procedure in real-life settings. First, because the present classification rates are very high, they need to be replicated. Extreme rates are often viewed with suspicion (see Meijer et al., 2013). Yet, recent deception research has consistently yielded very high accuracy rates. Rather than an anomaly, these outcomes are the result of a recent change in thinking that has led to changes in research design and focus (Levine, 2015). Indeed, some features of the current procedure may be responsible for the high classification rates, particularly guilty participants having to convince the interviewers they had performed some activities they had only general information about.

Second, one may argue that rates would be lower had we tested the predictive power of the cues with guilty and innocent participants other than those from whom the cues were derived, or had we tested the logistic function with a different sample. Please note, however, that we ran the logistic regression analyses both without and with cross-validation (with the leave-one-out method). Classification rates were the same regardless of whether cross validation was used for all analyses but one, and the drop in classification rates for this exceptional case was very small (see note b in **Table 2**). These findings indicate that the logistic models were very robust and likely generalizable. Moreover, our predictions were based on cognitive processes and the functioning of human memory, which is very similar across all healthy and normal individuals. Therefore, similar rates can be expected with different samples. Nonetheless, we strongly believe replication is important because the leave-one-out method does not substitute for a real cross validation.

Third, human judgments may differ from computer-based statistical classification. We are currently running a follow-up experiment in which human raters (both lay respondents and police officers) are given detailed instructions on how to code consistency and evasive answers, calculate the corresponding proportions for each suspect, and judge whether each suspect is lying or telling the truth using the empirically based cutoff points derived from the current data. If humans follow the instructions closely, identical classification rates as in the present study should be obtained. However, this issue needs to be examined empirically.

Fourth, if the current procedure is ever to be used in applied settings, practitioners would need to know what cutoff point they must use. In the current study, no guilty suspect gave the correct answer to more than 53% of questions, and no innocent participant did so to less than 65% of questions; thus, the optimal cutoff point to separate liars from truth tellers was located somewhere between 53 and 65%. For the combined variable, the optimal cutoff point was 73.33% (see **Figure 1**). However, cutoff points may differ across studies or situations. Nevertheless, the current response accuracy results are highly encouraging. They were likely due to guilty participants having little information about the truthful activities. This may not be specific of this study, but a feature shared by many false alibies. This issue needs to be empirically examined in future research. Please note that the cutoff issue affects most recent strategic interview approaches to detect deception; however, it has hardly (if at all) been discussed. We believe this issue needs to be considered before transferring laboratory-based techniques to field applications.

Fifth, in real life, special care should be taken concerning vulnerable suspects such as people with low IQ, highly suggestible, highly anxious, or with memory deficits. These personality characteristics may result in inaccurate, evasive or inconsistent answers even among truth tellers. Sixth, interview questions should presumably not involve false premises. Imagine there was no poster and an ill-informed interviewer asks about the location of the poster. In this case, replying “there was no poster” would not necessarily be an evasive answer used by guilty suspects to escape the situation, but could also be an honest reply given by an innocent suspect. The extent to which evasive answers are still useful to discriminate between liars and truth tellers when the questions involve false premises is a topic we are planning to examine in our future research.

Some additional caveats are in order. First, it can be argued that the tasks that the innocent participants performed were too artificial and different from normal real-life activities. A distinction has been made between mundane, experimental, and psychological realism (Aronson et al., 1998). *Mundane realism* refers to the extent to which the events occurring in the experimental context are likely to occur outside the laboratory (Aronson and Carlsmith, 1968), *experimental realism* refers to the extent to which the experimental session is involving to, and taken seriously by the participants (Aronson and Carlsmith, 1968), and *psychological realism* is the extent to which the psychological processes that play a role during the experiment are the same as those occurring outside the laboratory (Aronson et al., 1994). The hypotheses of the current study concerned the cognitive processes involved in encoding and retrieving central and peripheral information; thus, it was essential to place innocent suspects in a situation where they were exposed to central and peripheral details. However, it was irrelevant whether the situation was an artificial experimental session or a more real-life like event, because *the cognitive processes involved in encoding and retrieving central and peripheral information are the same in both kinds of contexts*. In other words, in this study psychological (and also experimental) realism were far more important than mundane realism. This does not limit the generalizability of the findings to more naturalistic situations inasmuch as the cognitive processes involved are the same (Aronson et al., 1998). Notwithstanding these arguments, we are currently designing more complex and ecologically valid studies to replicate the current findings in more naturalistic settings.

Second, the interview consisted of a number of closed questions. This is at odds with recommended strategies in investigative interviewing, which stress the importance of using open ended questions. The same can be said of polygraph tests and TRI-Con. However, it should be noted that the main purpose of all these procedures is to assess veracity rather than to collect abundant information from suspects.^8^ Nonetheless, the present procedure is compatible with the recommended techniques, which could be used afterward. It is also important to note that we are not suggesting that extant approaches should be replaced with the current procedure (not even with an improved and refined version of it). Instead, we believe that different situations call for different interview approaches. The procedure we began to explore could eventually be added to the interviewer’s toolbox to be used whenever alibi information is available from other sources and the suspect’s veracity is an issue.

Third, one can argue that the artificiality of the present procedure (focused questions, quick replies…) may lead suspects to refuse to cooperate (perhaps advised by their solicitors), or the courts not to accept the evidence obtained this way. Again, the same objections could be raised concerning the polygraph test, which is nevertheless used in the US by law enforcement agencies, the legal community, government agencies, and the private sector (American Polygraph Association, 2010). The polygraph admissibility in court varies across jurisdictions (American Polygraph Association, 2010). The polygraph is also used in criminal cases in Japan (Osugi, 2011). We see no reason why solicitors, the courts or the potential examinee would favor polygraph testing while objecting to our procedure. Other researchers defend the real-life application of even more artificial lie-detection methods, such as event-related potentials (Iacono, 2015). Other novel interviewing procedures, like TRI-Con (Walczyk et al., 2005, 2009, 2012) or reaction-time tests (Verschuere and De Houwer, 2011), are also similarly, “artificial.” Besides, presumably innocent suspects would not object to a procedure able to prove their innocence. However, before using new methods in real settings, replication studies are necessary, as well as ecologically valid research examining the limits of the procedures and potential countermeasures (Blandón-Gitlin et al., 2014). Furthermore, interviewers and the courts must be sensitive that refusal is not indicative of deception *per se*, and that one approach does not fit all: other approaches can be designed that generate no reluctance at all from suspects to cooperate.

Fourth, Interview 1 and Interview 2 were run by different interviewers. Critics may argue that this may have led suspects not to care about being consistent in the second interview. This is unlikely. The suspects knew the interviews were videotaped; thus, the second interviewer could know what the suspect’s answers had been during the first interview. More importantly, if a second interview is conducted, a reasonable inference is that the answers are going to be compared with those given during the first interview. Therefore, if anything, suspects may have tried hard to be consistent rather than not caring about consistency. In fact, our data question the notion that suspects did not care about being consistent, as both truth tellers’ (89% consistency) and liars’ (71% consistency) responses were considerably consistent across interviews. However, future research should replicate the current findings using the same interviewer across the two interviews.^9^

Fifth, both central and peripheral questions were asked during the interviews. It is a tenet in the memory literature that recall accuracy is better for central than for peripheral details (e.g., Ibabe and Sporer, 2004). This was also the case in the current study. However, in order to avoid floor effects, we selected peripheral questions for which recall in a preliminary study had been relatively high (though still significantly lower than recall for central information; see Appendix 2 in supplementary online materials). The present procedure may not work if the interview contains questions about peripheral details that innocent suspects failed to notice or encode. More specifically, if truth tellers did not encode peripheral details, one cannot expect them to give accurate, consistent, and non-evasive answers when asked about these details (see the polygraph literature for a similar concern regarding the *Concealed Information Test*; e.g., Meijer et al., 2016). If practitioners are to use this procedure in the future, they should select for the interview only those pieces of information most likely to have been noticed.^10^ Yet, note that the guilt status effect on both accurate answers and the combined variable was the same as strong regardless of question type. For inconsistencies, it was stronger for central than for peripheral questions. Therefore, practitioners would be well advised to use these diagnostic variables (in particular response accuracy and the combined variable) and to ask questions about noticeable *central* details only.

Finally, critics may argue that suspects interviewed by the police and then released may expect to be interviewed again. We know of no empirical research examining this issue. Until empirical data are available, any assumption either way is unwarranted. It is also possible that if the current procedure is ever used regularly in applied settings, then offenders might know, they may expect to be interviewed twice, and they may rehearse during the retention interval between the first and the second interview. Note, however, that these circumstances would potentially limit the use of inconsistencies but not the use of evasive answers, which discriminated between liars and truth tellers both during Interview 1 and during Interview 2. Therefore, running just one interview might suffice to use evasive answers as a deception cue. Besides, the current procedure cannot be employed in all cases; thus, there would be no reason for a specific suspect to assume that it would be used in his/her case. Notwithstanding these arguments, research should be conducted exploring the vulnerability of this interview procedure to countermeasures.

## Conclusions

Despite the above caveats and limitations, we believe the current findings are encouraging. We expect the present findings to replicate, and the procedure to be refined for its potential use in applied settings. Take, for instance, the case described at the beginning of this article. Both Kaled and Kuecken confessed to the crime but later recanted. Several individuals claimed that, at the time of the crime, the suspects were attending a party. The police could have interviewed those individuals to collect central and peripheral details about the party. A number of focused questions could then have been created about those details recalled by most of these individuals. Then, the police could have interviewed Kaled and Kuecken using the procedures described in this article. This way, maybe a miscarriage of justice would have been avoided.

## Author Contributions

JM conceived the research, supervised all tasks and procedures, analyzed the data and drafted the manuscript. JM, IB-G, and II formulated the hypotheses and designed the experiment and the procedures. JM and IB-G managed and supervised the data coding procedures. CM contributed to the design of the experimental procedures, ran all participants, entered all data, and checked the transcripts. JM, IBG, CH, and II interpreted the results. All five authors critically revised and eventually approved the manuscript.

## Conflict of Interest Statement

The authors declare that the research was conducted in the absence of any commercial or financial relationships that could be construed as a potential conflict of interest.

## Footnotes

1 This example is modeled after a real case in which the defendant himself had been in police custody at a different police station at the time of the crime. He was eventually convicted for a crime he could not have committed (see Schulte, 2013).

2 The reason why guilty participants were run first was that most of the hypotheses were based on the notion that guilty suspects would have little information about the innocent tasks. As described in the text, we took measures to prevent participants from sharing information about the study, but we nevertheless adopted the essential precaution of running the uninformed guilty participants first.

3 We initially recruited 50 suspects, and five suspects were scheduled to participate on every day. The five suspects of a day were all to be interviewed by the same interviewer. Thus, each interviewer was expected to interview five participants, and only 20 interviewers would be needed for all 50 participants to be interviewed twice. However, we needed to replace three suspects because of technical problems during Interview 1; this involved scheduling new sessions and recruiting additional interviewers. Further, two suspects did not follow the instructions and had therefore to be removed from the dataset.

4 The total number of questions asked by guilty participants was 215. However, unexpectedly, Coder 1 failed to code four questions and Coder 2 failed to code one question. In all, the number of questions coded by both coders was 210 (98% of all questions). These omissions were discussed afterward in the meeting the two coders held to resolve discrepancies.

5 All of this material is available from the first author on request.

6 As it is clear from the percentages, the difference between the number of central and peripheral questions was significant, χ^2^(1) = 182.50, *p* < 0.001. A Wilcoxon matched-pairs signed-ranked test was run with the participants (*n* = 24) as the unit of analysis. The test also indicated that participants asked significantly more questions about central than about peripheral information, *z* = –4.29, *p* < 0.001. Support for Hypothesis 1 was also found considering the separate ratings of each coder: Coder 1 coded 91.94% of questions as central, 1.90% as peripheral, and 6.16% as irrelevant. Coder 2 coded 91.59% of questions as central, 0.47% as peripheral, and 7.94% as irrelevant.

7 This interaction revealed that whereas accuracy in answering to central questions was higher in Interview 1, *M* = 0.72, *SD* = 0.31, than in Interview 2, *M* = 0.68, *SD* = 0.31, *p* = 0.015, *d* = 0.14, 95% CI [0.03,0.24], accuracy in answering to peripheral questions was similar across both interviews; for Interview 1: *M* = 0.46, *SD* = 0.30; for Interview 2: *M* = 0.48, *SD* = 0.32; *p* = 0.294, *d* = –0.06, 95% CI [–0.17,0.05]. Please note, however, that effect sizes were very small in both cases.

8 In reality, information about the alibi must be collected *before* using the current approach, either via tangible evidence or from other human sources (alibi witnesses). Also, unlike the polygraph Comparison Question Test, which focuses only on detecting deception, the Concealed Information Test can also be used to uncover new information (e.g., Verschuere and Meijer, 2014); however, it is its potential to establish guilt (by showing that the suspect has “concealed information”) that has been stressed by both researchers and practitioners.

9 A recent study by Shaw et al. (2014) found that switching interviewers increased inconsistencies in liars. However, their experimental paradigm was entirely different from the present one, which makes both studies hardly comparable. While we examined consistency *across repeated* interviews, Shaw et al. (2014) examined consistency *within the same* interview. Specifically, in Shaw et al.’s (2014) paradigm, the interviewers simply left before finishing their job, being replaced by new interviewers who completed the interview. Shaw et al. (2014) argued that because truth tellers realized that the new interviewers had not heard what they said earlier in the interview, they felt inclined to be detailed and to repeat what they had said. This explanation cannot be transferred to our paradigm: we argued above that, because suspects were videotaped, during Interview 2 they could infer that the second interviewer could compare their answers with those provided 1 week earlier during Interview 1. Shaw et al. (2014) also suggested that, for liars, the idea that their answers would be compared was less obvious than it was for truth tellers. It is unclear to us why this should be the case. Our parading also differed from Shaw et al.’s (2014) in terms of type of event, type of questions, type of response required, and—importantly—the way consistency was operationalized. The cognitive operations involved in each paradigm [Shaw et al. (2014) vs. ours] may differ. As a result, the outcomes of the two studies are not comparable (feel free contact the first author for a more detailed account of the differences). It is more likely that our findings were produced by the interview paradigm we used—which was designed precisely to produce these findings—than by merely switching the interviewer.

10 For example, if information about the alibi is collected from alibi witnesses, only those pieces of information recalled by most witnesses should be targeted during the interview.
