# Supplementary material for: Strategic Interviewing to Detect Deception: Cues to Deception across Repeated Interviews
Source: Front Psychol. 2016 Nov 1;7:1702. doi: 10.3389/fpsyg.2016.01702 (PMC5088571; doi:10.3389/fpsyg.2016.01702)
Supplement: Supplementary file 1 [file Data_Sheet_1.docx]

# Appendix 1: Interview Questions

Questions 1 and 2 were not scored. Questions 4, 6, 7, 9, 10, 13, 15, and 16 were central, and Questions 3, 5, 8, 11, 12, 14, 17, and 18 were peripheral.

1. Did you steal a wallet in Seminar Room 126? [All suspects expected to say “no”]

2. Then you have done some tasks in an office room with a researcher? [All suspects expected to say “yes”]

3. In what floor was that office room?

4. How many tasks did the researcher instruct you to do?

5. How many chairs were in the office room?

6. What was the second task?

7. What was the fourth task?

8. When you entered the room, what piece of music was playing over the speakers of the computer you would use to do the tasks?

9. What did you have to search in the Internet?

10. Where in the Internet did you have to search for it?

11. There was a poster in one wall; in which wall?

12. What character was in the poster?

13. What kind of arithmetic did you have to do?

14. Which was the background color of the screensaver in the computer you used to do the tasks?

15. What did you have to do with the information you retrieved from the Internet?

16. What questions were you asked about the documentary?

17. What book was on top of the computer keyboard?

18. What color was the door of the office room where you performed the tasks?

# Appendix 2: Preliminary Study

In the preliminary study, 21 undergraduate participants (*M* _age_ = 22.67 years, *SD* = 1.74) first performed the tasks of the innocent suspects and then completed two questionnaires. In the first questionnaire (*centrality questionnaire*), the definition and examples of central and peripheral questions were given to participants (see the definitions provided in this report). Then, the participants had to assess 54 questions about the event (24 designed to be peripheral, and 30 designed to be central) on a 1 (*peripheral*) to 6 (*central*) scale. In the second questionnaire (*accuracy questionnaire*), the participants had to reply in writing to each question.

The data collected with the centrality questionnaire allowed us to select two well-differentiated sets of questions, one of central and one of peripheral questions. Centrality scores were significantly higher for the eight selected central questions, *M* = 5.44, *SD* = 0.55, than for the eight selected peripheral questions, *M* = 2.46, *SD* = 1.21, *t* (20) = 10.09, *p* < .001, *d* = 3.17, 95% CI [2.03, 4.31].

The data collected with the accuracy questionnaire allowed us to select peripheral questions focusing on details that had been noticed by the [innocent] participants. This was done in order to prevent a floor effect for peripheral questions to occur—i.e., extremely low accuracy in replying to peripheral questions among both guilty and innocent suspects. Thus, accuracy (proportion of correct answers) in replying to the selected peripheral questions was fairly high, *M* = .82, *SD* = .19, but still significantly lower than accuracy in replying to the selected central questions, *M* = .97, *SD* = .06, *t* (20) = -3.83, *p* = .001, *d* = -1.08, 95% CI [-1.72, -0.44].
